# Waking Up Buried Memories of Old TV Programs

**DOI:** 10.3389/fnbeh.2017.00060

**Published:** 2017-04-10

**Authors:** Christelle Larzabal, Nadège Bacon-Macé, Sophie Muratot, Simon J. Thorpe

**Affiliations:** ^1^Centre de Recherche Cerveau et Cognition, Université de Toulouse, Université Paul SabatierToulouse, France; ^2^Centre National de la Recherche ScientifiqueCerCo, Toulouse, France

**Keywords:** very long-term memory, reactivations, re-consolidation, dormant memory

## Abstract

Although it has been demonstrated that visual and auditory stimuli can be recalled decades after the initial exposure, previous studies have generally not ruled out the possibility that the material may have been seen or heard during the intervening period. Evidence shows that reactivations of a long-term memory trace play a role in its update and maintenance. In the case of remote or very long-term memories, it is most likely that these reactivations are triggered by the actual re-exposure to the stimulus. In this study we decided to explore whether it is possible to recall stimuli that could not have been re-experienced in the intervening period. We tested the ability of French participants (*N* = 34, 31 female) to recall 50 TV programs broadcast on average for the last time 44 years ago (from the 60's and early 70's). Potential recall was elicited by the presentation of short audiovisual excerpts of these TV programs. The absence of potential re-exposure to the material was strictly controlled by selecting TV programs that have never been rebroadcast and were not available in the public domain. Our results show that six TV programs were particularly well identified on average across the 34 participants with a median percentage of 71.7% (*SD* = 13.6, range: 48.5–87.9%). We also obtained 50 single case reports with associated information about the viewing of 23 TV programs including the 6 previous ones. More strikingly, for two cases, retrieval of the title was made spontaneously without the need of a four-proposition choice. These results suggest that re-exposures to the stimuli are not necessary to maintain a memory for a lifetime. These new findings raise fundamental questions about the underlying mechanisms used by the brain to store these very old sensory memories.

## Introduction

As adults, we all have memories of sounds and images that were formed decades ago. For people who are now in their 70s and 80s, these memories are part of their very long-term memory also called remote memories. Memories can be semantic if they reflect general knowledge such as the ability to retrieve a movie title or episodic if they involve the recollection of a unique specific event in space and time: for example remembering a particular day when someone watched a movie on their first date (Tulving, 1985). When related to the self, both semantic and episodic memories create autobiographical memories that are specific to each individual. Therefore, autobiographical memories can involve generic facts about personal past events (“I used to watch my favorite TV series with my brother”) that are not always episodic (Tulving et al., 1988; Levine et al., 2002; Piolino et al., 2002).

Important neuronal reorganizations are required to create long-lasting memories and involve two consolidation stages (Frankland and Bontempi, 2005). The first one which is called synaptic consolidation refers to the stabilization of synaptic weights of a new memory in localized networks. This process is fast and can be completed within a few hours after learning. The second one, involving system-level consolidation, is much slower and corresponds to a change of brain regions that support the memory. Both the hippocampus and the neocortical structures are initially involved in supporting declarative memories (semantic and episodic memories). However, within several months after learning, theories suggest that declarative memories might become independent of the hippocampus. This would concern semantic and episodic memories in light of the so-called standard model (Squire and Alvarez, 1995) or simply semantic information regarding the Multiple Trace Theory (Nadel and Moscovitch, 1997). Note that the debate is still not closed between these two theories.

Given the practical difficulties involved, only a small number of studies have tried to test recall decades after the acquisition phase. Such studies have looked at memories concerning old classmates (Bahrick et al., 1975), information learnt in school (Bahrick, 1984; Conway et al., 1991), or even TV programs (Squire and Slater, 1975; Squire and Fox, 1980; Squire, 1989). Although the stimuli used are different, the results follow the same trend: recall drops quickly over the first 6 years and then levels off for several decades.

As mentioned by the authors, in such studies, one parameter that is hard to control fully is potential reactivations of the information during the intervening period which might explain the extremely long retention of these memories. It has been shown that memory reactivations can be triggered spontaneously during periods when the processing of sensory input is very low (“off-line states”) such as during sleep (Wilson and McNaughton, 1994; Peigneux et al., 2004; Diekelmann and Born, 2010) or wakefulness (Foster and Wilson, 2006; Peigneux et al., 2006; Karlsson and Frank, 2009; Oudiette et al., 2013) as well as during “on-line states” when subjects are actively retrieving memories (Nyberg et al., 2000; Gelbard-Sagiv et al., 2008; Tayler et al., 2013). While reactivations during “off-line states” are critical for the consolidation of a new memory (Gais et al., 2000; Girardeau et al., 2009) and are very frequent in the first few hours after learning (Ribeiro et al., 2004; Eschenko et al., 2008), their probability of occurrence is likely to decrease exponentially over time (McClelland et al., 1995; Frankland and Bontempi, 2005), leaving the memory in a dormant state (Lewis, 1979; Sara, 2000; Dudai, 2004). This suggests that the reactivation of these dormant or inactive memories might occur spontaneously, especially during “on-line” states when sensory input is strong enough to elicit memory retrieval, that is, during the re-exposure to the stimulus or when it is mentally evoked. For about 15 years now and since the discovery of Nader et al. (2000) it has been shown that reactivations of stable and consolidated memories through specific stimulus re-exposure might trigger re-consolidation processes. During this temporary unstable stage, memory traces are updated (Dudai, 2006) and strengthened (Moscovitch et al., 2006; Lee, 2008) and as a result, become more accessible and are less vulnerable to decay (Gisquet-Verrier and Riccio, 2012). Accordingly, it might be natural to think that remote memories could be maintained via subsequent re-consolidations that are triggered by specific stimuli, even if these re- consolidations are scattered in time. This leaves open the question of whether it is possible to retrieve very long-term memories for stimuli that have not been re-experienced for decades and that are left in a dormant state in the absence of any subsequent reactivations.

In this study we tested the ability of French people to recall a selected set of 50 TV programs that were originally broadcast with between 6 and 120 episodes between the late 50's and early 70's. These TV programs have never been rebroadcast and are not available in the public domain so we can be certain that the stimuli have not been seen or heard since the original broadcast. Furthermore, the participants reported that they had not thought about them for years, making it unlikely that they would have involuntarily reactivated the memories (Rasmussen and Berntsen, 2009).

By sorting participants performance over the confidence in their response, we found 6 TV programs with a percentage of correct identification (median: 71.7%, *SD* = 13.6, range: 48.5–87.9%) that was significantly higher than for younger participants. Interestingly, these 6 TV programs were part of a set of 23 videos for which we collected single case reports with associated information about the viewing at the time of broadcast. Whereas, identification was mainly performed by selecting the correct title from four propositions, in two cases, the presentation of short excerpts of old opening themes was able to trigger spontaneous naming of the title. Overall our data suggests that visual and auditory memories can indeed be retrieved even when they have been buried for decades.

## Materials and methods

### Participants

Thirty four subjects, all French (31 female; range = 52–92 years, median age = 77 years, mean age = 79 years, *SD* = 7.6) with normal or corrected-to-normal vision and audition participated in the study. All of these participants were recruited and tested individually in senior citizen clubs. Before starting the experiment participants were invited to respond to a questionnaire to give personal details about their TV habits. They were asked to say roughly when their household first had a television and how many hours a day they typically watched TV at the time of the test (Table S1). The participants reported first having a television sometime between 1956 and 1970 with half of them having access to a television before 1964 (*SD* = 4.1) and said that they currently were watching TV for an average of 3.5 h a day (range 0.5–9 h, *SD* = 2.1). After the experiment, participants gave their feedback about the TV programs they were able to recall. None of them reported thinking about the ones which had never been rebroadcast. The overall cognitive abilities of 25 out of the 34 older participants were also assessed using the Mini-Mental State Examination (MMSE) (Folstein et al., 1975) based on the French GRECO consensual version. No main deficit was found for any of the participants (mean score 27.6 out of 30, *SD* = 1.9, range: 24–30).

Thirty-four younger participants, [*t*_(66)_ = −32.0, *p* < 0.001], all French (23 female; range = 21–40 years, median age = 26 years, mean age = 27 years, *SD* = 5.5) with normal or corrected-to-normal vision and audition participated in the same study. Younger participants reported having access to a TV set from between 1976 and 1999 (mean year = 1992, *SD* = 5.9) and watching TV for 1.2 h a day (range = 0–5 h, *SD* = 1.5).

### Stimuli

Audiovisual clips (size: 640 ^*^ 480) were presented on a gray background at the center of a laptop screen placed in front of the participant (Hewlett Packard EliteBook, screen resolution: 1,366 ^*^ 768). The clips used a 1 s count-down followed by a 7 s opening theme of a TV program. The clips generally displayed the first 7 s of the opening themes without any text. In total 72 audiovisual clips were shown to each participant. Fifty of the clips were **test videos** that were composed of the opening theme excerpts of French TV programs which had never been rebroadcast and were not available in the public domain. With support from the French Audiovisual National Institute (INA), the videos were collected directly from an up-to-date database which has been collecting information on audiovisual programs broadcast since 1947. Videos were selected because they seemed original and because they did not change from the first to the last episode. The number of episodes was known for 36 of the 50 clips, and ranged from a minimum of 6 episodes to a maximum of 120, with a mean of 23.7. One of the programs was broadcast in the late 50's, 30 in the 60's and 19 were first broadcast in the 70's. The average year of the last episode broadcast was 1970. The remaining 22 clips were **famous videos** of the opening themes of well-known TV programs which in some cases were still on air and were not necessarily French (Table S2). They were supposed to be easy to recognize and to keep participants' interest during the task.

### Task

**Figure 1:**
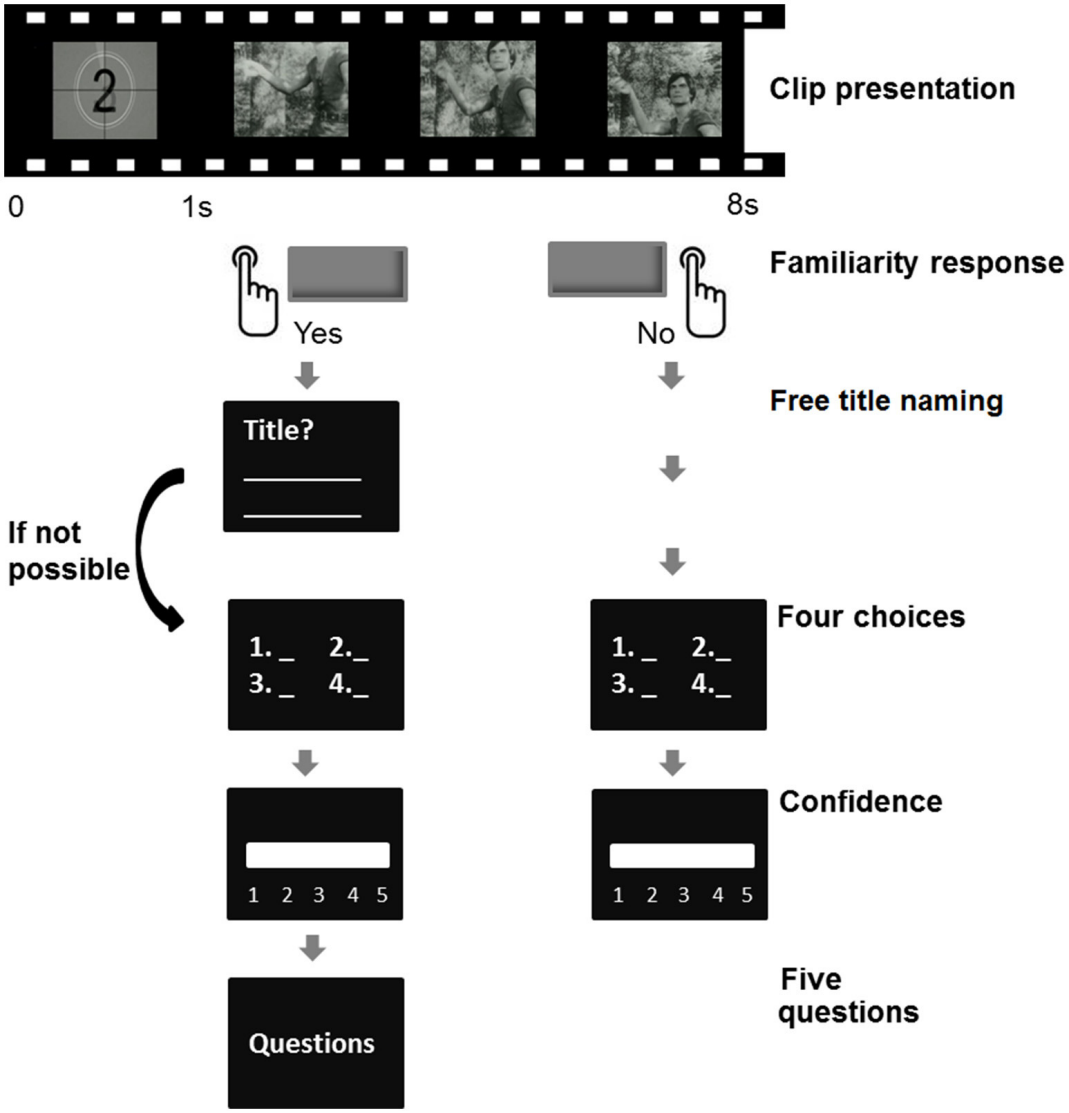
**Experimental design**. Audiovisual clips of the TV programs were shown on a computer screen. Participants decided whether the program was familiar by using a button press response. If familiar, participants attempted to give the title by free naming. Alternatively, they were given a four-proposition forced-choice (4-FC). The confidence level in the response was assessed on a five-point scale. For the familiar videos, five questions were asked to get associated information about the TV program.

The experiment involved 72 trials with each video clip being presented only once. Each trial started with the presentation of an 8-s video clip (Figure 1). During the video or after its display, participants' recall responses were collected as follows: (1) Does this TV program look familiar to you (Yes/No)? (2) If yes, can you name the title of this TV program? (Free title naming). If not, please choose the related-title from the four propositions in the forced-choice (4-FC). The four propositions included the correct title, a lure (title of another TV program) and two foils (fabricated titles). The lures and foils were selected to be as plausible as possible. Propositions were displayed in alphabetical order. (3) Rate your confidence in your response on a five-point scale (1: “Not sure at all”; 2: “A bit sure”; 3: “Fairly sure”; 4: “Very sure”; 5: “Completely sure”). Five questions were also asked when participants reported being familiar with the TV program in order to get associated information: (1) What day(s) of the week did you watch this TV program? (2) Around what time of the day: in the morning, in the afternoon or in the evening? (3) How old were you at that time? (4) Did you like this TV program? and (5) Give as much information as you can about this TV program (e.g.,: Who did you usually watch this TV program with? Have you watched a lot of episodes? What details could you give about the characters? etc.). Short breaks were made every 10 trials. The experiment lasted about 1 h and was programmed with Psychopy (Peirce, 2007).

In addition to the 34 participants tested, 34 younger participants performed the same task to make sure that the four titles proposed in the 4-FC would have the same probability of being chosen by naïve subjects who could only rely on semantic information to make their choice. Although it would have been possible to use age-matched controls, this was not the case here. We chose to test younger participants so that we could be certain that all the test videos were new to them. This would have been more difficult to control for older participants even if they said that they did not have a TV set in their house during that period, because at that time, it was relatively common to watch TV in someone else's home.

## Results

### Participants' performance for the famous TV programs

During the presentation of the audiovisual clips or after their display, participants were first invited to decide whether the TV program was familiar or not. The older participants reported that they were familiar with 56.5% (*SD* = 24.8) of the 22 famous audiovisual clips that were presented.

When participants said that a video was familiar, they were invited to name the title of the TV program directly (free title naming). On average, each famous video was spontaneously and correctly named by nine out of 34 older participants (27.5%, *SD* = 19.4, range = 0–25). This shows that the free naming of a title was high for the 22 famous TV programs and was always associated with a high confidence level (average = 4.5 up to 5, *SD* = 0.5). Overall, a median number of 6 (*SD* = 4.3) famous videos were correctly named spontaneously by the older participants, which was significantly lower than the 12 (*SD* = 4.9) correct titles reported spontaneously by the younger participants for the same famous videos (Wilcoxon signed-rank test: *Z* = −4.18, *p* < 0.001).

If participants found that it was too difficult to retrieve a title spontaneously, they were asked to select a title from four propositions (4-FC). When the older participants reported that they were familiar with the famous TV programs, the percentage of correct responses in the 4-FC was high (86.6%, *SD* = 21.7) and was not different from the younger participants (91.5%, *SD* = 14.9). The 4-FC was also directly proposed to the older participants who said that a video was not familiar. Overall, 68.8% (*SD* = 20.0) of the responses for famous clips were collected using the 4-FC including both familiar and unfamiliar responses. The older participants' performance was high when they had to choose the title of famous audiovisual clips (74.7%, *SD* = 15.5) and was not different from the younger participants (72.8%, *SD* = 12.9).

Interestingly, we found that the average percentage of identification for the famous videos was significantly correlated with the older participants' average confidence on the response (*r* = 0.71, *p* < 0.001, Pearson's linear correlation coefficient). The same effect was found in the younger participants (*r* = 0.79, *p* < 0.001, Pearson's linear correlation coefficient).

Overall, the older participants' performance was high for the 22 famous videos (median = 81.8%, *SD* = 9.7, range: 54.5–95.4%) showing that they could correctly perform the task. However, with a median of 90.9% (*SD* = 9.1, range: 54.5–95.4%) the younger participants' performance on the same videos was even better (Wilcoxon signed-rank test: *Z* = −3.37, *p* < 0.001). Such high levels of performance are explained by the fact that these famous TV programs are still very present in the media or even on TV. To what extent are the older participants able to identify old TV programs that have not been re-experienced for decades? We now address this question.

### Older participants' performance for the test TV programs

#### Single case reports for familiar test videos

On average, the older participants reported that they were familiar with 15.2% (*SD* = 13.1) of the test videos.

##### Free title naming for test videos

From the 50 test videos presented to 34 older participants, the titles of two TV programs were spontaneously recalled (free title naming). Although this percentage is very small (0.1%) it is still above the null score obtained by the 34 younger participants that were expected not to know the TV programs. Interestingly, these two free title namings were associated with a medium/high level of confidence in addition to associated information about the TV program. The following is a detailed description of the two responses:

On the first free recall, participant 5 (Table S1) said “Balzac,” which was close to the correct title “Un grand amour de Balzac.” Her confidence level was 3 out of 5. She was then asked to give details about her memories: “I was watching it on Saturday afternoon. I was sixty. Yes I've always loved sentimental and historical TV serials. I remember cousin Bette. I watched a lot of episodes.”

Actual facts: Episodes were broadcast in 1973 (she was 49 years-old, she is now 90) every Thursday at 9 p.m. Seven episodes of 52 min were screened.

The other participant, participant 22 (Table S1) said “Camember” for “Les facéties du sapeur Camember,” with a confidence response at 4 out of 5.

The participant then reported: “I used to watch it on Sunday evening. I was 35. I liked it but I did not watch it too often. The whole family used to watch it.”

Actual facts: Episodes were broadcast in 1965 (he was 28 years-old, he is now 77), every day, except on Sunday. The TV program had 50 episodes lasting 5 min each. Initially the program started at 8.30 p.m. but switched to 9 p.m. from the 20th episode.

##### 4-FC for familiar test videos

When participants reported to be familiar with a TV program but could not name it spontaneously they were asked to select a title from four propositions (4-FC). Overall the percentage of responses was 23.7% (*SD* = 24.8) for the correct titles, 43.8% (*SD* = 27.2) for the lures and 14.6% (*SD* = 16.7) and 17.9% (*SD* = 16.1) for the two foils (fabricated titles). For familiar famous videos, 9.0% (*SD* = 13.9) of the responses were attributed to lures and 4.4% (*SD* = 17.9) and 0% (*SD* = 0) to the two foils. This revealed that the older participants were significantly biased toward the lures for the test videos that were associated with a familiarity judgment, an effect that was not present for the famous videos [two-way ANOVA, ranked data: videos: *F*_(1, 236)_ = 23.06, *p* < 0.001, titles: *F*_(3, 236)_ = 60.81, *p* < 0.001, videos ^*^ titles: *F*_(3, 236)_ = 46.42, *p* < 0.001, *post-hoc* comparison using the Tukey-Kramer test].

Although the older participants were not above chance level on average across the 50 test videos we decided to analyze participants' performance for each TV program. Indeed, given the design of our experiment, we do not know whether the participants watched the whole 50 test TV programs and to what extent. We found that for 23 out of the 24 test TV programs that were correctly identified on the 4-FC associated information about the TV program was provided. On average these videos were reported 2.2 times (*SD* = 1.4) by 23 participants with a maximum of six times for “Un grand amour de Balzac.” Overall, the older participants were then able to report associated information for 2.8% (*SD* = 3.3) of the 50 old TV programs. Those responses were followed by a mean confidence level of 2.1 (*SD* = 0.9) which was significantly lower than the confidence for the correct free namings (mean: 3.5, *SD* = 0.7) but significantly higher than the confidence for the correct responses in the 4-FC for test videos judged unfamiliar (mean: 1.6, *SD* = 0.5) [*H*_(2)_ = 10.8, *p* < 0.01, *post-hoc* comparisons using the Tukey-Kramer test]. These 48 reports in addition to the 2 reports associated with free namings are given in the Table 1. Here are a few examples:

**Table 1:** **Collection of 50 reports for the test videos judged familiar by the older participants**.

	**Report (n°)**	**Participant (n°)**	**Confidence (/5)**	**What day?**	**What time?**	**How old?**	**Age during broadcast**	**Did you like it?**	**Other details**
**Un grand amour de Balzac (1973)** *Sentimental serial* 7 episodes Thursday, 9 p.m.	1	5	3	Saturday	Afternoon	60	49	Yes, I always loved sentimental and historical TV serials	I remember cousin Bette with Balzac. I watched a lot of episodes
	2	17	2	Every day	Afternoon	50	37	Yes	I watched it alone, a lot of episodes
	3	14	1	Weekdays	Evening	50	36	Yes	I watched it with my husband, rarely
	4	8	3	X	Evening	75	47	Yes	I watched it alone
	5	2	2	X	Evening	35	43	Yes	I watched it alone
	6	29	2	Weekdays	Evening	X	43	X	I have never watched it
**Poker d'as (1973)** *Spy serial* 26 episodes from Monday–Friday, 8 p.m.	7	25	2	Weekdays	Evening	50	33	Yes	I watched it with the whole family, not too often, before 1976 in Le Tarn Et Garonne (*French department*), I was 36
	8	13	2	Weekdays	Evening	30-35	37	Yes	I watched several episodes with my husband but without the children
	9	11	1	Monday	Evening	40	35	Not much	I watched it with my husband, very rarely, maybe one episode
	10	23	1	X	X	X	30	Not much	I watched it alone
**L'Homme de l'ombre (1968)** *Police drama* 30 episodes from Monday– Friday, 7.40 p.m.	11	12	2	Weekdays	Evening	43	26	A bit	In the bar
	12	23	1	X	Evening	37	25	A bit	Alone
	13	4	3	X	x	45	24	x	I have never watched it, only heard it
	14	15	2	X	x	45	29	Not much	x
**Frédéric le gardian (1965)** *Adventure series* 24 episodes From Monday to Sunday, Time: *unknown*	15	17	2	Weekdays	Evening	26	29	X	I watched it a few times with my husband
	16	8	2	X	Evening	75	39	Yes	X
	17	4	3	X	X	45	21	X	I have never watched it but I heard it in the bar
	18	20	2	X	X	X	34	X	X
**Bayard (1964)** *Youth series* 13 episodes Every Thursday, 6 p.m.	19	13	2	Wednesday or Thursday	Afternoon	40	28	Yes a lot	The children liked it a lot, we watched all the episodes
	20	4	2	X	We were in the bar	20	20	X	I remember hearing the music
	21	15	2	X	X	35	25	No	I watched it with my husband
**Champion (1964-1966)** *Game show* Episodes: *unknown* Tuesday, 8.50 p.m.	22	17	2	Every day	Evening	50	28-30	Yes	I watched it with my husband, we watched a lot of episodes
	23	13	1	X	X	40	28	X	I maybe watched it with my husband
	24	15	2	X	X	X	25	Not much	X
**Courte Echelle (1974)** *Youth TV program* ~100 episodes From Monday to Saturday, 6.35 p.m.	25	8	3	X	Afternoon	70	48	Yes	I watched it often and alone
	26	32	5	X	X	X	33	Yes	I don't know if the whole family watched it but I watched it every time
	27	24	3	X	X	X	52	X	X
**L'arche de Samsong (1972)** *Youth TV program (mini musical play)* 37 episodes From Monday to Saturday, 3.20 p.m.	28	30	1	Weekdays	Evening	30	34	Not very much	I watched it a bit with the whole family
	29	14	1	X	X	X	35	X	I have never seen it but the music reminds me of something
	30	13	1	X	X	X	36	X	X
**Le train bleu s'arrête 13 fois (1965-1966)** *Police drama* 13 episodes Friday every 2 weeks 9.35 p.m. (or 22.35 p.m. for the 4th episode)	31	14	1	Saturday	Afternoon	35	28	Yes	I watched it occasionally with the children, when we were in the house in Toulouse
	32	30	2	Weekdays	Evening	35	27	Yes	I watched it with the whole family, occasionally
**Les facéties du sapeur Camember (1965)** *Adventure series* 50 episodes Every day except Sunday 8.30 p.m. for the 1st to the 20th episode	33	22	4	Sunday	Evening	35	28	Yes	The whole family used to watch it but not too often
	34	8	3	X	Evening	60	39	Yes	I watched it alone
**Teuf-Teuf (1968-1969)** *Youth game show* ~111 episodes From Monday to Saturday, about 6 p.m.	35	17	3	Wednesday	Evening	30	32	Yes	I watched a lot of episodes with the children
	36	4	2	X	X	I was young or older	24	X	I only listened to the music, I have never watched it
**Vol 272 (1964)** *Drama series* 13 episodes Every Sunday, 7.25 p.m.	37	14	1	Weekdays	X	X	27	X	I have never watched it
	38	4	3	X	X	X	20	X	It was a long time ago, I heard it in the bar
**Miss (1979)** *Detective series* 6 episodes Day ? Time?	39	12	2	Weekdays	Evening	63	37	Yes	I occasionally watched it in the bar
	40	23	2	X	X	35	36	A bit	X
**En direct avec (1966-1968)** *Political TV program* Monthly Day? Evening	41	30	3	Weekdays	Evening	30	28-30	No	I watched it a few times, with the whole family
**Encore un carreau de cassé (1960-1961)** *Youth game show* Episodes, day and time: unknown	42	12	2	Wednesday	Evening	37	18	A bit	I was in the bar, I watched it a lot
**La boîte à malice (1979)** *Youth game show* Episodes? Day? Afternoon	43	11	2	Wednesday	Afternoon	40	41	No	I watched it with my children when we were in Montauban (*French town*)
**Le roi qui vient du sud (1979)** *historical serial*6 episodes, Thursday, 8.30 p.m.	44	17	2	Weekend	Afternoon	30	43	Yes	I watched a lot of episodes with the whole family
**Que ferait donc Faber? (1969)** *Comedy series* 8 episodes, Thursday, 9.40 p.m.	45	7	2	Weekdays	Evening	50-62	46	No	We watched it by chance. We watched a few episodes when we were in Castres (*French town*)
**Animal Parade (1972)** *Youth series* 12 episodes Every day, 7.30 p.m.	46	16	3	Weekdays and weekend	Afternoon	X	41	Yes	I watched it with the children
**Candice ce n'est pas sérieux (1969)** *Comedy series* 20 episodes Every day, 1.20 p.m.	47	13	2	X	Afternoon or evening	40	33	I think so	I watched it with my children and my husband. I don't know if i watched a lot of episodes or not
**Commandant X (1962)** *Spy serial* 10 episodes, Day: unkown, 8.50 p.m.	48	27	1	X	Afternoon	30	22	Yes	48
**Alexandre Bis (1974)** *Spy serial* 6 episodes, Thursday, 8.50 p.m.	49	33	2	X	Evening	About 50	49	Yes	X
**Objectif Demain (1979-1981)** *Scientific TV program* Episodes and day: unknown, 8.45 p.m.	50	4	2	X	Evening	45	35	X	I watched it without paying much attention
**La vérité sur l'espionnage (1967)** *Adventures series* 13 episodes Monday, 10.30 p.m.	0	14	1	X	X	X	30	X	I have never watched it

*The 2 reports: report 1 and 33 were made after spontaneous retrieval of the correct title. The other 48 reports were given after correctly choosing the title in the 4-FC. The columns: “What day?,” “What time?,” “How old,” “Like?” and “Other details” correspond respectively to the responses to the 5 questions asked to get associated information about the TV programs. The column “Age during broadcast” was the actual age of the participant during the broadcast of the TV program*.

Report 8 (Table 1): After the presentation of the TV program “Poker d'as,” participant 13 (Table S1) reported that she was familiar with the TV program and chose the correct title on the 4-FC with a confidence rate of 2 out of 5 (“a bit sure,” average confidence for all test TV programs: 1.3, *SD* = 0.4). Then she reported that she liked the TV program and watched it during the week, in the evening, when she was 30–35. She added that she used to watch it with her husband but without the children and remembered having seen several episodes.

Actual facts: “Poker d'as” was a one-season TV series of 26 episodes broadcast in 1973 from Monday to Friday at 8 p.m. The participant was 37 at that time and was 78 when tested.

Report 46 (Table 1): Participant 16 (Table S1) reported she was familiar with “Animal Parade” and correctly identified it in the 4-FC. Her confidence rate was 3 (“medium sure,” average confidence for the 50 test TV programs: 1.4, *SD* = 0.8). Then she reported watching it during the week and at weekends in the afternoon. She could not remember how old she was at the time but she remembered that she liked it and watched it with her children.

Actual facts: “Animal Parade” was a single-season youth TV program broadcast from the 14th to the 25th of February (week and weekend) in 1972 at 7.30 p.m.

Report 45 (Table 1) is interesting because of the 21 famous and test videos considered as familiar by participant 7 (Table S1), she reported that there were only two TV programs she did not like: “30 millions d'amis” an animal TV magazine and “Que ferait donc Faber?,” a comedy series broadcast in 1969. We found that there had been a large controversy following the broadcast of “Que ferait donc Faber?” with a lot of criticism from the French newspapers and the viewers. Other details reported by participant 7 (who was 91 when tested) and in particular the day and time of broadcast or her age matched the actual facts.

Among the 34 participants that were recruited we had the opportunity to test a couple individually on the same day: participants 25 and 30 (Table S1) who have had a TV set since 1964. Interestingly, participant 25 reported being familiar with “Poker d'as” and chose the correct title in the 4-FC with a confidence rate of two (“a bit sure,” average confidence for the 50 test TV programs: 1.0, *SD* = 0.1). He said that he used to watch it on weekdays during the evening when he was fifty. He added that he liked the TV program and watched it with his family but not too often. This was when they were still in Tarn-et-Garonne (*a department in the South of France*) a bit before 1976 so when he was 36 (report 7, Table 1). Interestingly by recollecting this last piece of information, participant 7 corrected himself concerning his age at the time of broadcast. And indeed, this TV program was broadcast on weekdays at 8 p.m. in 1976 when he was 33. Unlike her husband participant 30 did not report any familiarity with “Poker d'as” but picked the correct title from the 4-FC with a confidence rate of 3 out of 5 (“medium sure,” average confidence for the 50 test TV programs: 1.5, *SD* = 0.7).

A detailed analysis of the older participants' performance shows that the number of famous and test TV programs for which associated information was reported ranged between 1 and 23 (mean = 12.8, *SD* = 5.3) and were negatively and linearly correlated with participants age (*r* = −0.49, *p* < 0.05, Pearson's correlation coefficient). The three male participants who were tested (participants 12, 22, and 25) were ranked in the top half.

Concerning the test TV programs, 10 of the older participants were able to give associated information for at least two TV programs, 12 others for only one TV program, whereas the remaining 12 participants were not able to give associated information for any of the test TV programs including the youngest and oldest participants (participants 19 and 24).

These limited examples suggest that it is indeed possible to recall memories of TV programs that were not re-experienced for decades. Moreover, our data suggest that retrieval can be associated with subjective familiarity, some confidence in the response and with information about the viewing of the TV programs. Although it is hard to verify fully the accuracy of the associated information reported by the participants (such as the fact that participants liked the TV program or used to watch it with specific relatives), some information such as participants' age or the days and time of broadcast could give an idea of the accuracy of the memory. However, as suggested by the last report (report 50), the memory of old TV programs might not always be associated with direct familiarity judgment. In the following section we analyzed the data on the 4-FC by including the TV programs considered as not familiar.

#### Older participants' performance for each test video

Most of the older participants responses were collected via the 4-FC: 97.4% (*SD* = 3.5) for the test TV programs. On average, the older participants were 24.4% (*SD* = 5.7) correct in identifying the title of a test video, which is similar to the younger participants: 22.9% (*SD* = 5.3) that were expected to perform at chance level in this task (25%). It is important to notice that in our study and as opposed to classical recall experiments we do not know whether all the 50 test videos were watched by every participant and to what extent. We therefore analyzed the older participants' performance on the 4-FC for every audiovisual clip.

**Figure 2:**
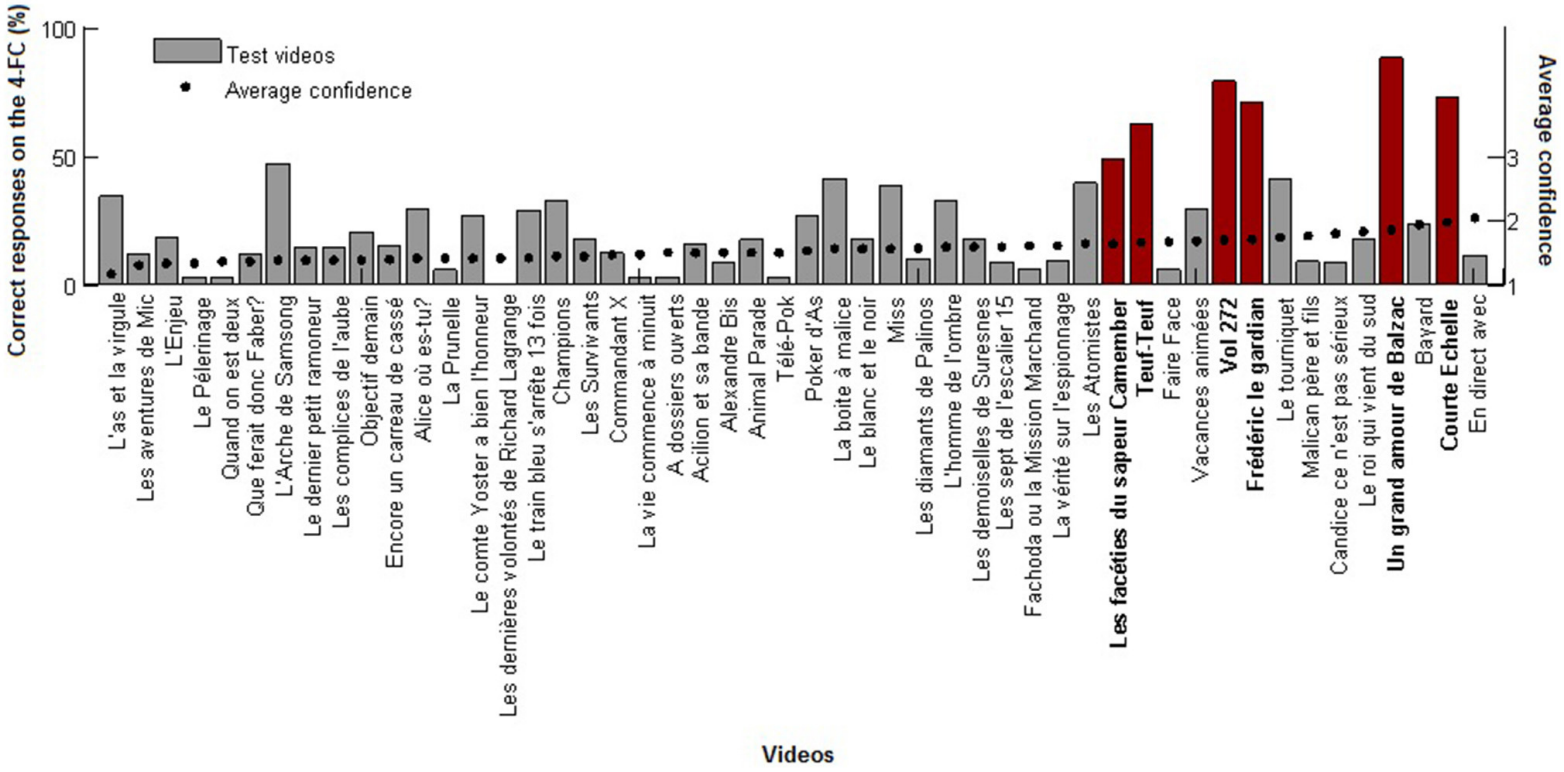
**Performance in the 4-FC for the 50 test TV programs**. Videos were sorted according to the average confidence in the responses given by the older participants in the 4-FC for videos judged familiar or not. The older participants' performance for the six TV programs are shown in red: “Les facéties du sapeur Camember,” “Teuf-Teuf,” “Vol 272,” “Frédéric le gardian,” “Un grand amour de Balzac” and “Courte Echelle.”

Interestingly, we found that the average percentage of identification of the test videos was significantly correlated with the average confidence on the response (*r* = 0.37, *p* < 0.01, Pearson's linear correlation coefficient) which was not the case for the younger participants (*r* = 0.06, Pearson's linear correlation coefficient). As shown in Figure 2, by sorting the average performance of each test video over the average confidence in the response, six titles were particularly well identified by the older participants when they used the 4-FC, namely: “Les Facéties du Sapeur Camember” (16 participants out of 33), “Teuf-Teuf” (20 participants out of 32), “Vol 272” (26 participants out of 33), “Frédéric le Gardian” (24 participants out of 34), “Un Grand Amour de Balzac” (29 participants out of 33) and “Courte Echelle” (24 participants out of 33). The median percentage of correct identification for these six TV programs was high: 71.7% (*SD* = 13.6, range: 48.5–87.9%) and significantly different from the 33.8% (*SD* = 20.6, range: 8.8–61.8%) obtained by the younger participants from the same six TV programs (Wilcoxon Signed-rank test: *Z* = 21, *p* < 0.05). Note that all of these titles were also found in the reports presented in Table 1, including “Les Facéties du Sapeur Camember” and “Un grand Amour de Balzac” which were spontaneously named.

### Influencing factors

In this experiment several factors might explain the variability in the older participants' performance for the test videos. This might be due to variations between the audiovisual clips or to individual differences.

In particular it would be interesting to find out if there was something about the 23 TV programs out of 50 that were associated with some contextual information, compared with the 27 that failed to work with anyone. This could be explained by the number of broadcasts, the overall duration of a TV program (episode duration ^*^ number) or the time interval since the last episode was broadcast. However, for each of these three factors we did not find any difference between the 23 TV programs recognized and the other 27.

Another source of variability might concern the audiovisual content presented during the 7 s of each clip. In particular, some of the videos were more static than others. We therefore decided to count the number of different scenes defined as a specific action in space and time for each of the test videos (mean = 1.6, *SD* = 1.1, range: 1–7). We did not find any difference between the number of scenes displayed in the 23 test videos that were recognized compared to the other 27.

The older participants' performance for the 50 test videos might also be related to inter-individual differences. In particular we tested for the impact of five factors: age, cognitive abilities (given by the MMSE score), the year of TV set acquisition, the number of hours participants currently watched TV and the performance obtained for the 22 famous TV programs. We found that the percentage of correct responses for the 50 test videos was negatively and linearly correlated with participants' age (*r* = −0.39, *p* < 0.05, Pearson's correlation coefficient) and with the year of TV set acquisition (*r* = −0.40, *p* < 0.05, Pearson's correlation coefficient). However, we did not find any correlation between participants' performance for the 50 test videos and these following three factors: (1) the time the older participants spent watching TV when tested, (2) the older participants' performance for the famous videos, (3) the older participants' overall cognitive abilities. For this latter factor, only 25 participants were tested.

## Discussion

This study shows that recalling a complex audiovisual stimulus is possible decades after the original exposure, under conditions where reactivation in the intervening period is very unlikely. The absence of re-exposure to the stimuli was strictly controlled by testing TV programs that have never been rebroadcast and are not available in the public domain. However, we cannot completely rule out the possibility that reactivation might occur during exposure to content that was indirectly related to these TV programs. Nor can we totally exclude involuntarily reactivations that might happen during sleep or wakefulness (Rasmussen and Berntsen, 2009; Rubin and Berntsen, 2009). Indeed, the stimuli used in this study are rich in terms of content and could elicit semantic associations with other items that are not directly related to the TV programs for example. Further experiments should use abstract/meaningless or simple stimuli to rule out this hypothesis. Nevertheless, given the absence of physical re-exposure to the stimuli and the fact that participants reported not having thought about these TV programs for years, the probability of occurrence of these reactivations might be very small when considering the retention interval between the time of broadcast and the recall. This gives enough support to consider that the memories of these old TV programs were dormant or in an inactive state.

Interestingly, all of the information retrieved concerned generic information about the test TV programs. They could be personal memories (participants' age, whether they liked the TV program or not, who they used to watch the TV program with and when) or not (title or characters' name of the TV program). The amount of personal details retrieved from autobiographical memory varied but even for the most detailed reports, no reference to a specific or unique event was mentioned. Several factors could explain participants' inability to retrieve any episodic autobiographical memory: the age (Rubin and Schulkind, 1997) and long retention interval between watching the TV programs and doing the test (Piolino et al., 2002) as well as the contextual similarity in which the episodes of the same TV program were watched. In the latter case, each episode of a TV program would be a new episode from which participants would create a generic pattern stored semantically (Neisser, 1981).

The retrieval of semantic autobiographical memory was associated with familiarity together with some confidence in the response. This shows that familiarity might be a property of the semantic system, a recent view that emerged from lesion studies (Vargha-Khadem et al., 1997). If this is the case, the recognition of a TV program was based on a judgment of previous occurrence performed consciously by the participant (Mandler, 1980). However, and as shown by the six TV programs that were identified with better than chance level performance over the 34 participants, most of the recognized test TV titles occurred without any familiarity judgment and with low confidence levels. In this case, title recognition might only be due to an effect of perceptual fluency of which the participants were unaware of Jacoby and Dallas (1981), Verfaellie and Cermak (1999), and Voss et al. (2008). Participants might select a title because they could process it more readily given the video presented. Judgments based on perceptual fluency alone might not be the most optimal (Jacoby and Dallas, 1981) but could explain the bias toward lures for the test TV programs.

In our experiment, 23 out of the 50 test TV programs were able to elicit the retrieval of semantic autobiographical memories. We would have expected that a difference in the number of original broadcasts would have an impact on the retrieval of personal contextual details. However, we did not find any difference between these 23 TV programs and the other 27. Indeed, as shown by reports 1–5 and 45 (Table 1) it seems that only a few exposures to a TV series or its opening theme (report 6, Table 1) can be enough to create a stable memory trace. We also thought that the time elapsed since the last broadcast could be a critical factor, but again we did not find any difference between the 23 TV programs that elicited retrieval of personal details and the other 27. Nevertheless, the chance of getting any recall for these 23 TV programs was higher than it was for the two oldest TV programs broadcast in the late 50's: “Les Aventures de Mic” and “Télé-Pok,” for which no report was collected and for which correct identification on the 4-FC was respectively 12.1 and 2.9%.

Interestingly, the median age of the participants at the time when these 23 TV programs were broadcast was 33 years old (*SD* = 8.4, range 18–52) which corresponds to a critical period in the developmental literature: adults above 35 years old are able to retrieve many autobiographical memories during this period of early adulthood (Rybash, 1999; Rathbone et al., 2008). This might have had an impact on the older participants' ability to recall the test TV programs in semantic memory.

This age effect was confirmed when considering participants' overall performance for the 50 test TV programs. Indeed we found that the best performers were the youngest subjects from the older group of participants.

To get access to the participants' memories of old TV programs, this study was carried-out in an ecological way in comparison to classical lab-based memory paradigms. However, these real-life conditions lead to two main limitations. (1) The population we tested was heavily biased toward females, with only three males among the 34 participants. This bias is largely explained by differences in participants' willingness to be involved in this study, together with a difference of mean life expectancy between the genders in France (French male: 80 years, French female: 85 years). The small number of male participants made it impossible to find significant performance differences between the genders, but further studies would be useful.

(2) We don't know whether the 50 test TV programs were watched by all the participants tested. However, we found that the earlier participants had a television in their house the better their overall performance was for the test videos.

The biggest question raised by this study concerns the nature of the underlying mechanisms of these very long-term memories. How is it possible that such dormant memory traces can survive for decades, even when there is no possibility of re-exposure to the original stimuli? So far, studies have mainly focused on the role of re-consolidation in the maintenance of long-term memory. Findings show that reactivations of long-term memories re-consolidate and strengthen memory traces and for episodic memories this occurs with the support of a more distributed ensemble of hippocampal–neocortical neurons (Moscovitch et al., 2006). Because the TV programs used had between 6 and 120 episodes, such re-consolidations might have occurred during the initial period of broadcast. At the same time, the retrieval of similar episodic events during the original broadcasting would allow the transformation of episodic information into semantic representations corresponding to a gist or schema created from all the episodes watched for the same TV program (Winocur et al., 2010; Winocur and Moscovitch, 2011). For one TV program, an episodic memory of each TV episode would then coexist with a generic semantic representation of all its episodes. According to this Trace Transformation Theory that follows the Multiple Trace Theory, episodic memories would rely on both neocortical and hippocampal structures whereas the semantic representation would be specific to the neocortex.

In the absence of reactivations after the original broadcasting, the synaptic weights of the neurons supporting these memories—in the hippocampus and/or in the neocortex—would not have been much reinforced. And so far, little is known concerning the maintenance of these dormant memories. It is hard to conceive the preservation of these synaptic weights over time when considering a very large population of neurons as the support of the memory trace. In such networks the constant incoming of new inputs might interfere with previous memories that tend to be overwritten (Hopfield, 1982; Gardner, 1987). We suggest that the initial consolidation phase that enables the formation of a stable memory trace could be associated with the increased selectivity of a small number of neocortical neurons. This selectivity might rely on a simple STDP (Spike Time Dependent Plasticity) mechanism that allows simulated neurons to become selective to arbitrary input patterns if they occur repeatedly (Masquelier and Thorpe, 2007; Bichler et al., 2011; Klampfl and Maass, 2013) and even after a small number of repetitions (Andrillon et al., 2015). One of the characteristics of this sort of spike based learning is that synaptic weights are only modified if the target neuron fires (Markram et al., 1997; D'amour and Froemke, 2015). Selective neurons, that are not firing for any new incoming stimuli, would therefore be able to keep memories for a long time (Thorpe, 2011) in a silent way until their excitatory-inhibitory balance might be disrupted (Barron et al., 2016). It has been suggested that all of these inactive cortical neurons might form a kind of “dark matter” in the brain (Binzegger et al., 2004; Shoham et al., 2006; Thorpe, 2011). Re-exposure to a specific stimulus might elicit the firing of the otherwise inactive neurons which might trigger its recall and potentially the recollection of contextual specifics through binding in the hippocampus.

Our paradigm used audiovisual clips to reactivate these dormant memories and it is quite possible that such stimuli could be more efficient than static and unimodal stimuli (Furman et al., 2007). We strongly believe that the use of dynamic and multimodal stimuli should be more widespread and might provide valuable assistance in the diagnosis of memory loss or impairment in conditions such as Alzheimer's disease. We are open to providing the audiovisual clips to researchers interested in using the material.

It is as yet unclear how such long-term memories can be retained over decades and it should be noted that synaptic plasticity may not be the only possibility. For example, there is evidence that some forms of memory can be transmitted epigenetically (Crick, 1984; Carone et al., 2010; Dias and Ressler, 2014) although it seems unlikely that such mechanisms could be involved in memories for old TV themes. We hope that further investigations will be carried out to understand the “life” of these dormant memories.

## Ethics statement

All participants gave written informed consent prior to experimentation. The protocol was carried out in accordance with guidelines approved by the Ethics Evaluation Committee of Inserm (CEEI).

## Author contributions

Conceptualization and methodology: CL, NB, and ST.; Investigation: CL and SM.; Writing: CL, NB, and ST; Funding acquisition: ST; Supervision: NB and ST.

### Conflict of interest statement

The authors declare that the research was conducted in the absence of any commercial or financial relationships that could be construed as a potential conflict of interest.

## References

[B1] Andrillon T. Kouider S. Pressnitzer D. Agus T. (2015). Perceptual learning of acoustic noise generates memory-evoked potentials. Curr. Biol. 25, 1–7. 10.1016/j.cub.2015.09.027 26455302

[B2] Bahrick H. P. (1984). Semantic memory content in permastore: fifty years of memory for spanish learned in school. J. Exp. Psychol. 113, 1–29. 10.1037/0096-3445.113.1.1 6242406

[B3] Bahrick H. P. Bahrick P. O. Wittlinger R. P. (1975). Fifty years of memory for names and faces: a cross-sectional approach. J. Exp. Psychol. Gen. 104, 54–75. 10.1037/0096-3445.104.1.54

[B4] Barron H. C. Vogels T. P. Emir U. E. Makin T. R. O'Shea J. Clare S.. (2016). Unmasking latent inhibitory connections in human cortex to reveal dormant cortical memories. Neuron 90, 191–203. 10.1016/j.neuron.2016.02.031 26996082 PMC4826438

[B5] Bichler O. Querlioz D. Thorpe S. J. Bourgoin J.-P. Gamrat C. (2011). Unsupervised features extraction from asynchronous silicon retina through Spike-Timing-Dependent Plasticity, in Proceedings of the IEEE International Joint Conference on Neural Networks 859–866. 10.1109/IJCNN.2011.6033311

[B6] Binzegger T. Douglas R. J. Martin K. A. (2004). A quantitative map of the circuit of cat primary visual cortex. J. Neurosci. 24, 8441–8453. 10.1523/JNEUROSCI.1400-04.2004 15456817 PMC6729898

[B7] Carone B. R. Fauquier L. Habib N. Shea J. M. Hart C. E. Li R.. (2010). Paternally-induced transgenerational environmental reprogramming of metabolic gene expression in mammals. Cell 143, 1084–1096. 10.1016/j.cell.2010.12.008 21183072 PMC3039484

[B8] Conway M. A. Cohen G. Stanhope N. (1991). On the very long-term retention of knowledge acquired through formal education : twelve years of cognitive psychology. J. Exp. Psychol. Gen. 120, 395–409. 10.1037/0096-3445.120.4.395

[B9] Crick F. (1984). Memory and molecular turnover. Nature 312, 101. 10.1038/312101a0 6504122

[B10] D'amour J. A. Froemke R. C. (2015). Inhibitory and excitatory spike-timing-dependent plasticity in the auditory cortex. Neuron 86, 514–528. 10.1016/j.neuron.2015.03.014 25843405 PMC4409545

[B11] Dias B. G. Ressler K. J. (2014). Parental olfactory experience influences behavior and neural structure in subsequent generations. Nat. Neurosci. 17, 89–96. 10.1038/nn.3594 24292232 PMC3923835

[B12] Diekelmann S. Born J. (2010). The memory function of sleep. Nat. Rev. Neurosci. 11, 114–126. 10.1038/nrn2762 20046194

[B13] Dudai Y. (2004). The neurobiology of consolidations, or, how stable is the engram? Annu. Rev. Psychol. 55, 51–86. 10.1146/annurev.psych.55.090902.142050 14744210

[B14] Dudai Y. (2006). Reconsolidation: the advantage of being refocused. Curr. Opin. Neurobiol. 16, 174–178. 10.1016/j.conb.2006.03.010 16563730

[B15] Eschenko O. Ramadan W. Mölle M. Sara S. J. (2008). Sustained increase in hippocampal sharp-wave ripple activity during slow-wave sleep after learning. Learn. Mem. 15, 222–228. 10.1101/lm.726008 18385477 PMC2327264

[B16] Folstein M. F. Folstein S. E. McHugh P. R. (1975). A practical state method for grading the cognitive state of patients for the clinician. J. Psychiat. Res. 12, 189–198. 10.1016/0022-3956(75)90026-6 1202204

[B17] Foster D. J. Wilson M. A. (2006). Reverse replay of behavioural sequences in hippocampal place cells during the awake state. Nature 440, 680–683. 10.1038/nature04587 16474382

[B18] Frankland P. W. Bontempi B. (2005). The organization of recent and remote memories. Nat. Rev. Neurosci. 6, 119–130. 10.1038/nrn1607 15685217

[B19] Furman O. Dorfman N. Hasson U. Davachi L. Dudai Y. (2007). They saw a movie: long-term memory for an extended audiovisual narrative. Learn. Mem. 14, 457–467. 10.1101/lm.550407 17562897 PMC1896095

[B20] Gais S. Plihal W. Wagner U. Born J. (2000). Early sleep triggers memory for early visual discrimination skills. Nat. Neurosci. 3, 1335–1339. 10.1038/81881 11100156

[B21] Gardner E. (1987). Maximum storage capacity in neural networks. Europhys. Lett. 4, 481–485. 10.1209/0295-5075/4/4/016

[B22] Gelbard-Sagiv H. Mukamel R. Harel M. (2008). Internally generated reactivation of single neurons in human hippocampus during free recall. Science 322, 96–101. 10.1126/science.1164685 18772395 PMC2650423

[B23] Girardeau G. Benchenane K. Wiener S. I. Buzsáki G. Zugaro M. B. (2009). Selective suppression of hippocampal ripples impairs spatial memory. Nat. Neurosci. 12, 1222–1223. 10.1038/nn.2384 19749750

[B24] Gisquet-Verrier P. Riccio D. C. (2012). Memory reactivation effects independent of reconsolidation. Learn. Mem. 19, 401–409. 10.1101/lm.026054.112 22904371

[B25] Hopfield J. J. (1982). Neural networks and physical systems with emergent collective computational abilities. Proc. Natl. Acad. Sci. U.S.A. 79, 2554–2558. 10.1073/pnas.79.8.2554 6953413 PMC346238

[B26] Jacoby L. L. Dallas M. (1981). On the relationship between autobiographical memory and perceptual learning. J. Exp. Psychol. Gen. 110, 306–340. 10.1037/0096-3445.110.3.306 6457080

[B27] Karlsson M. P. Frank L. M. (2009). Awake replay of remote experiences in the hippocampus. Nat. Neurosci. 12, 913–918. 10.1038/nn.2344 19525943 PMC2750914

[B28] Klampfl S. Maass W. (2013). Emergence of dynamic memory traces in cortical microcircuit models through STDP. J. Neurosci. 33, 11515–11529. 10.1523/JNEUROSCI.5044-12.2013 23843522 PMC6618695

[B29] Lee J. L. (2008). Memory reconsolidation mediates the strengthening of memories by additional learning. Nat. Neurosci. 11, 1264–1266. 10.1038/nn.2205 18849987

[B30] Levine B. Svoboda E. Hay J. F. Winocur G. Moscovitch M. (2002). Aging and autobiographical memory: dissociating episodic from semantic retrieval. Psychol. Aging 17, 677–689. 10.1037/0882-7974.17.4.677 12507363

[B31] Lewis D. J. (1979). Psychobiology of active and inactive memory. Psychol. Bull. 86, 1054–1083. 10.1037/0033-2909.86.5.1054 386401

[B32] Mandler G. (1980). Recognizing : the judgment of previous occurrence. Psychol. Rev. 87, 252–271. 10.1037/0033-295X.87.3.252

[B33] Markram H. Lübke J. Frotscher M. Sakmann B. (1997). Regulation of synaptic efficacy by coincidence of postsynaptic APs and EPSPs. Science 275, 1–4. 10.1126/science.275.5297.213 8985014

[B34] Masquelier T. Thorpe S. J. (2007). Unsupervised learning of visual features through spike timing dependent plasticity. PLoS Comput. Biol. 3:e31. 10.1371/journal.pcbi.0030031 17305422 PMC1797822

[B35] McClelland J. L. McNaughton B. L. O'Reilly R. C. (1995). Why there are complementary learning systems in the hippocampus and neocortex: insights from the successes and failures of connectionist models of learning and memory. Psychol. Rev. 102, 419–457. 10.1037/0033-295X.102.3.419 7624455

[B36] Moscovitch M. Nadel L. Winocur G. Gilboa A. Rosenbaum R. S. (2006). The cognitive neuroscience of remote episodic, semantic and spatial memory. Curr. Opin. Neurobiol. 16, 179–190. 10.1016/j.conb.2006.03.013 16564688

[B37] Nadel L. Moscovitch M. (1997). Memory consolidation, retrograde amnesia and the hippocampal complex. Curr. Opin. Neurobiol. 7, 217–227. 10.1016/S0959-4388(97)80010-4 9142752

[B38] Nader K. Schafe G. E. Le Doux J. E. (2000). Fear memories require protein synthesis in the amygdala for reconsolidation after retrieval. Nature 406, 722–726. 10.1038/35021052 10963596

[B39] Neisser U. (1981). John dean's memory: a case study. Cognition 9, 1–22. 10.1016/0010-0277(81)90011-1 7196816

[B40] Nyberg L. Habib R. McIntosh A. R. Tulving E. (2000). Reactivation of encoding-related brain activity during memory retrieval. Proc. Natl. Acad. Sci. U.S.A. 97, 11120–11124. 10.1073/pnas.97.20.11120 11005878 PMC27158

[B41] Oudiette D. Antony J. W. Creery J. D. Paller K. A. (2013). The role of memory reactivation during wakefulness and sleep in determining which memories endure. J. Neurosci. 33, 6672–6678. 10.1523/JNEUROSCI.5497-12.2013 23575863 PMC3677604

[B42] Peigneux P. Laureys S. Fuchs S. Collette F. Perrin F. Reggers J.. (2004). Are spatial memories strengthened in the human hippocampus during slow wave sleep? Neuron 44, 535–545. 10.1016/j.neuron.2004.10.007 15504332

[B43] Peigneux P. Orban P. Balteau E. Degueldre C. Luxen A. Laureys S. (2006). Offline persistence of memory-related cerebral activity during active wakefulness. PLoS Biol. 4:e100. 10.1371/journal.pbio.0040100 16602824 PMC1413571

[B44] Peirce J. W. (2007). PsychoPy–Psychophysics software in Python. J. Neurosci. Methods 162, 8–13. 10.1016/j.jneumeth.2006.11.017 17254636 PMC2018741

[B45] Piolino P. Desgranges B. Benali K. Eustache F. (2002). Episodic and semantic remote autobiographical memory in ageing. Memory 10, 239–257. 10.1080/09658210143000353 12097209

[B46] Rasmussen A. S. Berntsen D. (2009). The possible functions of involuntary autobiographical memories. Appl. Cogn. Psychol. 23, 1137–1152. 10.1002/acp.1615

[B47] Rathbone C. J. Moulin C. J. Conway M. A. (2008). Self-centered memories: the reminiscence bump and the self. Mem. Cogn. 36, 1403–1414. 10.3758/MC.36.8.1403 19015500

[B48] Ribeiro S. Gervasoni D. Soares E. S. Zhou Y. Lin S. C. Pantoja J. (2004). Long-lasting novelty-induced neuronal reverberation during slow-wave sleep in multiple forebrain areas. PLoS Biol. 2, 126–137. 10.1371/journal.pbio.0020024 14737198 PMC314474

[B49] Rubin D. C. Berntsen D. (2009). The frequency of voluntary and involuntary autobiographical memories across the life span. Mem. Cognit. 37, 679–688. 10.3758/37.5.679 19487759 PMC3044938

[B50] Rubin D. C. Schulkind M. D. (1997). The distribution of autobiographical memories across the lifespan. Mem. Cogn. 25, 859–866. 10.3758/BF03211330 9421572

[B51] Rybash J. (1999). Aging and autobiographical memory: the long and bumpy road. J. Adult Dev. 6, 1–10. 10.1023/A:1021667823176

[B52] Sara S. J. (2000). Retrieval and reconsolidation: toward a neurobiology of remembering. Learn. Mem. 7, 73–84. 10.1101/lm.7.2.73 10753974

[B53] Shoham S. O'Connor D. H. Segev R. (2006). How silent is the brain: is there a “dark matter” problem in neuroscience? J. Comp. Physiol. A 192, 777–784. 10.1007/s00359-006-0117-6 16550391

[B54] Squire L. R. (1989). On the course of forgetting in very long-term memory. J. Exp. Psychol. Learn. Mem. Cogn. 15, 241–245. 10.1037/0278-7393.15.2.241 2522513

[B55] Squire L. R. Alvarez P. (1995). Retrograde amnesia and memory consolidation: a neurobiological perspective. Curr. Opin. Neurobiol. 5, 169–177. 10.1016/0959-4388(95)80023-9 7620304

[B56] Squire L. R. Fox M. M. (1980). Assessment of remote memory : validation of the television test by repeated testing during a 7-year period. Behav. Res. Methods Instrum. 12, 583–586. 10.3758/BF03201847

[B57] Squire L. R. Slater P. C. (1975). Forgetting in very long-term memory as assessed by an improved questionnaire technique. J. Exp. Psychol. Hum. Learn. Mem. 104, 50–54. 10.1037/0278-7393.1.1.50

[B58] Tayler K. K. Tanaka K. Z. Reijmers L. G. Wiltgen B. J. (2013). Reactivation of neural ensembles during the retrieval of recent and remote memory. Curr. Biol. 23, 99–106. 10.1016/j.cub.2012.11.019 23246402

[B59] Thorpe S. J. (2011). Visual population codes, in Toward a Common Multivariate Framework for Cell Recording and Functional Imaging, eds Kriegeskorte N. Kreiman G. (Cambridge, MA: MIT Press), 23–51.

[B60] Tulving E. (1985). Memory and consciousness. Can. Psychol. Can. 26, 1–12. 10.1037/h0080017

[B61] Tulving E. Schacter D. L. McLachlan D. R. Moscovitch M. (1988). Priming of semantic autobiographical knowledge: a case study of retrograde amnesia. Brain Cogn. 8, 3–20. 10.1016/0278-2626(88)90035-8 3166816

[B62] Vargha-Khadem F. Gadian D. G. Watkins K. E. Connelly A. Van Paesschen W. Mishkin M. (1997). Differential effects of early hippocampal pathology on episodic and semantic memory. Science 277, 376–380. 10.1126/science.277.5324.376 9219696

[B63] Verfaellie M. Cermak L. S. (1999). Perceptual fluency as a cue for recognition judgments in amnesia. Neuropsychology 13, 198–205. 10.1037/0894-4105.13.2.198 10353371

[B64] Voss J. L. Baym C. L. Paller K. A. (2008). Accurate forced-choice recognition without awareness of memory retrieval. Learn. Mem. 15, 454–459. 10.1101/lm.971208 18519546 PMC2414256

[B65] Wilson M. A. McNaughton B. L. (1994). Reactivation of hippocampal ensemble memories during sleep. Science 265, 676–679. 10.1126/science.8036517 8036517

[B66] Winocur G. Moscovitch M. (2011). Memory transformation and systems consolidation. J. Int. Neuropsychol. Soc. 17, 766–780. 10.1017/S1355617711000683 21729403

[B67] Winocur G. Moscovitch M. Bontempi B. (2010). Memory formation and long-term retention in humans and animals: convergence towards a transformation account of hippocampal-neocortical interactions. Neuropsychologia 48, 2339–2356. 10.1016/j.neuropsychologia.2010.04.016 20430044

