# Supplementary material for: Waking Up Buried Memories of Old TV Programs
Source: Front Behav Neurosci. 2017 Apr 10;11:60. doi: 10.3389/fnbeh.2017.00060 (PMC5385357; doi:10.3389/fnbeh.2017.00060)
Supplement: Supplementary file 1 [file Table1.DOCX]

**Supplementary information**

| **Participant** | **Gender** | **Age when tested** | **Year of TV acquisition** | **Hours of TV/day** |
| --- | --- | --- | --- | --- |
| 1 | Female | 76 | 1956 | 3.5 |
| 2 | Female | 84 | 1960 | 2 |
| 3 | Female | 81 | 1970 | 5 |
| 4 | Female | 70 | 1958 | 1 |
| 5 | Female | 90 | 1959 | 2 |
| 6 | Female | 85 | 1963 | 6 |
| 7 | Female | 91 | 1958 | 6 |
| 8 | Female | 88 | 1967 | 3 |
| 9 | Female | 84 | 1958 | 3 |
| 10 | Female | 75 | 1963 | 4 |
| 11 | Female | 76 | 1962 | 1 |
| 12 | Male | 72 | 1956 | 3 |
| 13 | Female | 78 | 1965 | 4 |
| 14 | Female | 77 | 1963 | 0.5 |
| 15 | Female | 75 | 1962 | 1 |
| 16 | Female | 83 | 1960 | 9 |
| 17 | Female | 78 | 1957 | 3 |
| 18 | Female | 79 | 1963 | 2 |
| 19 | Female | 52 | 1964 | 2 |
| 20 | Female | 83 | 1970 | 5.25 |
| 21 | Female | 73 | 1965 | 2.5 |
| 22 | Male | 77 | 1963 | 3 |
| 23 | Female | 71 | 1968 | 4 |
| 24 | Female | 92 | 1966 | 1 |
| 25 | Male | 74 | 1964 | 3 |
| 26 | Female | 76 | 1970 | 2 |
| 27 | Female | 74 | 1966 | 7.5 |
| 28 | Female | 79 | 1968 | 6 |
| 29 | Female | 84 | 1965 | 8 |
| 30 | Female | 76 | 1964 | 5 |
| 31 | Female | 76 | 1967 | 3 |
| 32 | Female | 73 | 1965 | 2 |
| 33 | Female | 89 | 1967 | 2.5 |
| 34 | Female | 84 | 1970 | 4 |

**Table S1**: Older participants’ detailed information: gender, age, year of TV acquisition and daily number of hours of watching TV at the time of the recall.
