# Supplementary material for: Waking Up Buried Memories of Old TV Programs
Source: Front Behav Neurosci. 2017 Apr 10;11:60. doi: 10.3389/fnbeh.2017.00060 (PMC5385357; doi:10.3389/fnbeh.2017.00060)
Supplement: Supplementary file 2 [file Table2.DOCX]

**Supplementary information**

| **Title** | **Date 1st episode** | **Date last episode** | **Rebroadcast** | **Number of episodes** | **Episodes duration (min)** | **Number of seasons** |
| --- | --- | --- | --- | --- | --- | --- |
| **Famous TV programs** | | | | | | |
| 30 millions d'amis | 6 January 1976 | On the air the 1 January 2015 | No | > 1000 | 25 | 39 |
| Age tendre et tête de bois | 30 May 1961 | 1 April 2014 | No | ? | ? | ? |
| Agence tous risques | 1 July 1984 | 8 March 1987 | Yes | 98 | 45 | 5 |
| Alerte à Malibu | 2 January 1991 | 1 January 2002 | Yes | 243 | 45 | 11 |
| Bonne nuit les petits | 10 December 1962 | 1 December 1973 | Yes | 568 | 5 | 5 |
| Chapeau melon et bottes de cuir | 7 January 1961 | 21 May 1969 | Yes | 161 | 52 | 6 |
| Dallas | 2 April 1978 | 3 May 1991 | Yes | 357 | 45 | 14 |
| Des chiffres et des lettres | 4 January 1972 | On the air the 1 January 2015 | No | ? | ? | ? |
| Eurovision | 24 May 1956 | On the air the 1 January 2015 | No | 58 | 180 | ? |
| Fort boyard | 7 July 1990 | On the air the 1 January 2015 | No | 277 | 110 | 25 |
| Inspecteur Gadget | 24 October 1983 | 1 January 1986 | Yes | 86 | 22 | 2 |
| Intervilles | 17 July 1962 | On the air the 1 January 2015 | No | 250 | 120 | 24 |
| La petite maison dans la prairie | 30 March 1974 | 21 March 1983 | Yes | 205 | 45 | 9 |
| Mac Gyver | 29 September 1985 | 21 May 1992 | Yes | 139 | 45 | 7 |
| Magnum | 11 December 1980 | 1 May 1988 | Yes | 162 | 50 | 8 |
| Mission impossible | 17 September 1966 | 30 March 1973 | Yes | 171 | 50 | 7 |
| Motus | 25 June 1990 | On the air the 1 January 2015 | No | > 1000 | 30 | 24 |
| Stade2 | 28 December 1975 | On the air the 1 January 2015 | No | > 1000 | 70 | 39 |
| Starsky et Hutch | 30 April 1975 | 15 May 1979 | Yes | 93 | 48 | 4 |
| Thalassa | 27 September 1975 | On the air the 1 January 2015 | No | > 1000 | 60 | 37 |
| Thierry la fronde | 3 November 1963 | 27 March 1966 | No | 52 | 25 | 4 |
| Wonder Woman | 7 November 1975 | 11 September 1979 | Yes | 59 | 48 | 3 |
| **Test TV programs** | | | | | | |
| A dossiers ouverts | 18 February 1974 | 22 March 1974 | No | 25 | 13 | 1 |
| Acilion et sa bande | 3 July 1978 | 31 August 1980 | No | ? | ? | 2 |
| Alexandre Bis | 25 July 1974 | 5 September 1974 | No | 6 | 78 | 1 |
| Alice où es-tu? | 16 August 1969 | 8 September 1969 | No | 20 | 13 | 1 |
| Animal Parade | 14 February 1972 | 25 February 1972 | No | 12 | 7 | 1 |
| Bayard | 16 January 1964 | 9 April 1964 | No | 13 | 25 | 1 |
| Candice ce n'est pas sérieux | 9 September 1969 | 1 October 1969 | No | 20 | 13 | 1 |
| Champions | 21 May 1964 | 12 July 1966 | No | ? | ? | ? |
| Commandant X | 30 October 1962 | 17 July 1965 | No | 10 | 90 | 1 |
| Courte Echelle | 8 February 1974 | 8 July 1974 | No | ~100 | 150 | 1 |
| En direct avec | 3 October 1966 | 8 April 1968 | No | ? | ? | ? |
| Encore un carreau de cassé | 11 aout 1960 | 29 April 1961 | No | ? | ? | ? |
| Fachoda ou la mission Marchand | X March 1977 | 1 April 1977 | No | 6 | 60 | 1 |
| Faire Face | 10 June 1960 | 9 February 1962 | No | 11 | 30 | 2 |
| Frédéric le gardian | 23 September 1965 | 16 October 1965 | No | 24 | 14 | 1 |
| L'arche de Samsong | 8 November 1972 | 26 Decembrer 1972 | No | 37 | 15 | 1 |
| L'as et la virgule | 19 November 1964 | 23 December 1964 | No | 8 | 45 | ? |
| L'Enjeu | X September 1978 | 13 June 1988 | No | ? | ? | ? |
| L'homme de l'ombre | 29 July 1968 | 6 September 1968 | No | 30 | 13 | 1 |
| La Prunelle | 10 September 1968 | 3 December 1968 | No | 13 | 30 | 1 |
| La boîte à malice | 1 July 1979 | 30 August 1979 | No | ? | ? | ? |
| La vérité sur l'espionnage | 4 December 1967 | 1 January 1969 | No | 13 | 50 | ? |
| La vie commence à minuit | 28 July 1967 | 6 August 1967 | No | 20 | 13 | 1 |
| Le blanc et le noir | 14 January 1975 | 9 September 1975 | No | ? | ? | ? |
| Le comte Yoster a bien l’honneur | 30 July 1969 | 5 October 1974 | No | 40 | 25 | 2 |
| Le dernier petit ramoneur | 6 October 1961 | 1 January 1961 | No | ? | ? | ? |
| Le Pélerinage | 5 April 1975 | 5 May 1975 | No | 24 | 13 | 1 |
| Le roi qui vient du sud | 8 February 1979 | 15 March 1979 | No | 6 | 52 | 1 |
| Le Tourniquet | 1971 | 1971 | No | ? | ? | ? |
| Le train bleu s'arrête 13 fois | 8 October 1965 | 11 March 1966 | No | 90 | 13 | 1 |
| Les Atomistes | 12 February 1968 | 18 March 1968 | No | 26 | 13 | 1 |
| Les Survivants | 20 September 1965 | 6 October 1965 | No | 13 | 13 | 1 |
| Les Aventures de Mic | 2 April 1959 | 14 September 1960 | No | 52 | 15 | 1 |
| Les complices de l'aube | 1 September 1965 | 9 September 1965 | No | 9 | 15 | 1 |
| Les demoiselles de Suresnes | 15 April 1968 | 21 May 1968 | No | 26 | 13 | 1 |
| Les dernières volontés de Richard Lagrange | 4 July 1972 | 7 August 1972 | No | 30 | 13 | 1 |
| Les diamants de palinos | 31 December 1964 | 14 January 1965 | No | 13 | 13 | 1 |
| Les facéties du sapeur Camember | 20 September 1965 | 18 November 1965 | No | 50 | 5 | 1 |
| Les sept de l'escalier 15 | 3 January 1967 | 6 February 1967 | No | 25 | 13 | 1 |
| Malican père et fils | 15 July 1967 | 27 July 1967 | No | 13 | 26 | 1 |
| Miss | 26 July 1979 | 16 August 1979 | No | 6 | 55 | 1 |
| Objectif Demain | 19 March 1979 | 16 december 1981 | No | ? | ? | ? |
| Poker d'as | 31 October 1973 | 7 December 1973 | No | 26 | 13 | 1 |
| Quand on est deux | 18 November 1962 | 31 January 1964 | No | 26 | 13 | 1 |
| Que ferait donc Faber ? | 3 July 1969 | 21 August 1969 | No | 8 | 55 | 1 |
| Télé-Pok | 11 September 1957 | 11 February 1958 | No | ? | ? | ? |
| Teuf Teuf | 30 October 1968 | 7 March 1969 | No | ~111 | ? | 1 |
| Un grand amour de Balzac | 19 April 1973 | 31 May 1973 | No | 7 | 50 | ? |
| Vacances animées | 14 March 1974 | 1 January 1977 | No | 120 | 45 | 3 |
| Vol 272 | 3 May 1964 | 26 July 1964 | No | 13 | 26 | 1 |

**Table S2**: Details of famous and test TV programs: broadcast period, number of episodes, duration of episodes and number of seasons. For some TV programs the number of episodes was not known and an approximate numbers (~…) was calculated based on the days of broadcast and the dates of beginning and end of broadcast. Question marks represent missing data.
